# Attachment Styles, Personality Organization, and Substance Use as Predictors of Emotion Regulation Strategies “Suppression” and “Reappraisal” in Young Adults

**DOI:** 10.3389/fpsyt.2021.786045

**Published:** 2022-01-21

**Authors:** Pauline L. Burgkart, Xenia Vuzic, Jürgen Fuchshuber, Human-Friedrich Unterrainer

**Affiliations:** ^1^Department of Psychology, University of Graz, Graz, Austria; ^2^Center for Integrative Addiction Research (CIAR), Grüner Kreis Society, Vienna, Austria; ^3^Department of Philosophy, University of Vienna, Vienna, Austria; ^4^Department of Psychiatry and Psychotherapeutic Medicine, Medical University Graz, Graz, Austria; ^5^Department of Religious Studies, University of Vienna, Vienna, Austria

**Keywords:** attachment, personality organization, substance use, emotion regulation, young adults

## Abstract

**Background:**

As evidenced by current literature, there is a crucial link between emotion regulation, attachment, personality patterns, and substance abuse. However, knowledge regarding the exact interactions of these specific parameters in terms of substance abuse development is still sparse. Therefore, this study is aimed to shed light on how two specific emotion regulation strategies (“Reappraisal” and “Suppression”) might be influenced by the relationship between attachment, structural deficits in personality organization, and addictive behaviors.

**Method:**

A total sample of 299 non-clinical young adults (Age: M = 22; SD = 3.81; 74.2% females) filled in the Emotion Regulation Questionnaire (ERQ) together with the Adult Attachment Scale (AAS), the Personality Organization Inventory (IPO-16), and the Alcohol, Smoking and Substance Involvement Screening (ASSIST) by means of an online survey.

**Results:**

As suggested by hierarchical regression analysis, attachment specifically predicted differences in Emotion Regulation (ER), whereby the AAS subscales “Close” ness (β = −0.38, *p* < 0.01) and “Depend” ence (β = –0.18, *p* < 0.01) were negatively associated with increased use of maladaptive strategies of expressional suppression of emotion, and “Depend” (β = 0.26, *p* < 0.01) was positively associated with increased use of adaptive strategies of cognitive reappraisal.

**Discussion:**

In line with our assumptions, we observed a more secure attachment system to be predictive for an increased use of adequate emotion regulation strategies. The findings support the suggestion that a focus on underlying attachment-related processes in a psychotherapeutic setting might be a promising way to promote adaptive self-regulation of emotions.

## Introduction

Emotions are an indispensable component of living, as “they can direct attention to key features of the environment, optimize sensory intake, tune decision making, ready behavioral responses, facilitate social interactions, and enhance episodic memory” [(1), p. 3].

To live a functioning and fulfilling emotional life, one has to regulate their emotions. The process of Emotion Regulation (ER) is understood as a bundle of strategies and processes that change appearance, intensity, durability, and expression of emotion (2). Some of these strategies can be seen as adaptive, and some of them can be seen as maladaptive. The use of predominantly maladaptive strategies and the lack of adaptive strategies can be seen as risk factors for psychopathology (3). Furthermore, adaptive ER shows negative correlations with Neuroticism and anxious-depressive symptoms, whereas for maladaptive strategies, it works the other way around, and the selection of emotion regulation strategies shows in part a mediating role in the relationship between adolescents' Neuroticism and increased mood pathology (4).

In line with Gross and John (5) and in order to narrow down the relatively broad ER concept, mainly two specific ER strategies are addressed in this study. (1) *Cognitive reappraisal* (“Reappraisal”) describes antecedent-focused strategies of cognitively changing the appraisal of situations and therefore changing their emotional impact; (2) *Expressive suppression* (“Suppression”) means the response modulation of inhibiting the (mimic, verbal, or gestural) expression of one's emotion (6). What is more, “Reappraisal” is seen as an adaptive strategy, which leads to increased subjective well-being, and both affective and social functioning (5). “Suppression” of emotional expression on the other hand has more negative implications for affective and social functioning, as well as for subjective wellbeing (5, 6) and is less effective at modifying affect (7).

The basis for successful ER is connected to various variables, among which Object relations are to mention. Object relations mean the “internalization of significant relations between self and others as the fundamental building blocks of the mind” [(8), p. 41]. These internal representations of relationships influence various parts of our behavior in adulthood, among other things one's way to attach to others or to organize one's personality (8, 9).

Early positive interactions of a child with its caregiver allow a person to reasonably relate to one's environment and regulate one's affect on this secure basis, whereas early negative or traumatizing childhood experiences can lead to an impairment regarding the development of healthy internalized object relations. In turn, this results in decreased personality organization and a predominance of insecure attachment styles, which predict decreased emotion functioning in adult life (10). In correspondence to this, maltreated children search less for support of their mother while expressing emotions, report to be less likely to share their emotions with their mother, and have fewer coping strategies for anger at their disposal, which indicates that the experience of physical or psychological abuse may influence the emotional development (11). In general, experiences of abandonment or violence, especially at a young age, make it more difficult for individuals (also in adulthood) to “get through life with an affectively positive framework” [(12), p.3]. Correspondingly, Desatnik et al. (13) state that different variables of internal representations of relationships (IRR) formed in early childhood account for almost 50% of variation in ER. Furthermore, there is substantial evidence that successful ER is increased in individuals experiencing support from their close social environment (14). This shows that the actual attachment behavior in adulthood might change the use of different ER strategies, and therefore the developmental perspective of ER as an attachment-related variable is of high interest. A securely attached individual is more likely to have a functional social support system at their disposal than an insecure attached one. In line with this, insecure attachment was observed to be connected to more maladaptive ER strategies and impairment of neural structures and neural functioning (15). Considering the two strategies of ER (“Suppression” and “Reappraisal”) illuminated in this study, literature suggests that highly secure attached people tend to show a greater use of the adaptive strategy of “Reappraisal,” whereas insecure attached (fearful or avoidant) individuals reported greater use of the maladaptive strategy of expressional “Suppression” (16).

What is more, ER was found to be influenced by the level of personality organization, whereby a higher amount of personality disorganization was found to be linked to diminished ER capabilities (13). In line with social baseline theory (17), identity and coherence of self (ICS) could be linked to higher functioning ER; in detail, ICS predicted reduced activation in brain regions responsible for the intrinsic strategy of expressional “Suppression” indicating less effort and more efficacy during ER (13). Furthermore, there is evidence suggesting that ER strategies mediate the relationship between Big Five personality traits and psychotic experiences in a non-clinical sample (18). Additionally, the Big-5 personality factors “Extraversion” and “Openness” predict more “Reappraisal” use and all Big-5 personality factors predict less use of “Suppression,” except for “Neuroticism,” which leads to higher rates in the use of “Suppression” (19). Regarding structural deficits in “Personality Organization,” there is a noticeable connection to “Reappraisal” and “Suppression.” For example, individuals with high schizotypal traits show hindered “Reappraisal” and biased “Suppression” (20), while negative correlations were found for borderline symptomatology and “Reappraisal,” and positive correlations were found for borderline symptomatology and “Suppression” (21).

By expanding the study of addiction as a kind of attachment disorder to ER research, impaired ER can be seen as a crucial factor underlying the increased vulnerability to substance abuse (15). Thereby, from a developmental perspective, impoverished ER can be discussed as both the origin and the result of substance abuse [e.g., (22, 23)]. Correspondingly, higher rates of substance abuse as well as increased psychopathology were observed to be related to maladaptive or less effective styles of ER (24, 25). Therefore, various authors [e.g., (25)] argue that drugs are used to either increase positive emotions or alleviate negative emotional states, including anxiety, sadness, and depression, which again increases the likelihood of taking drugs by means of a positive feedback mechanism. Whereas, in the beginning, using drugs is perceived as an adaptive strategy to regulate one's emotions, in the long run, it can lead to addiction because of its reinforcing characteristics (26). Furthermore, a deficient attachment system and the isolation that comes along with it effectuate that people with insecure or fearful attachment patterns are not able to resort to intact inner representations of the self and others, which leads in turn to external use of substances to cope with difficult situations or disruptions in close relationships (27–30). Accordingly, on a neurochemical level, an overlap can be found between social attachment and drug addiction, in particular in the mesolimbic dopamine pathway (31). Correspondingly, poly-drug users show impairments regarding the reactivity within the white matter and reduced cortical thickness, which in turn is linked to more insecure attachment as well as to more negative affectivity (32, 33). Therefore, an attachment-specific side effect of substance abuse is the reduced exploration of one's inner world as a suppressing strategy. In correspondence to this, substance use might be understood as a substitute for lacking coping strategies regarding the handling of strong emotions (34, 35).

### Research Aims

Based on the established connection between increased addictive behaviors and attachment and personality dysfunctioning, we intend in this study to investigate the role of those parameters for the selection of ER strategies more in detail. It is hypothesized that higher attachment and personality pathology as well as substance use might lead to a higher difficulty to regulate negative and positive emotions.

## Methods

### Participants and Procedure

The participants were recruited by means of the University of Graz mail distribution system. After informing the participants of the anonymity of the study and declaring consent, each participant was asked to fill in the questionnaires *via* the online-survey platform LimeSurvey©. Participants were included if they filled in all questionnaires and were aged between 18 and 35 years. A total of 21 people were excluded because they did not meet the age criteria. In total, 299 people were included in the study. The study was carried out in accordance with the Declaration of Helsinki. Ethical approval was granted by the Ethics Committee of the University of Graz, Austria. The recruitment of participants was carried out between September 2020 and January 2021.

### Psychometric Instruments

#### Emotion Regulation

The *Emotions Regulation Questionnaire* (5) is a self-report questionnaire capturing two specific Emotion Regulation strategies in terms of dealing with positive and negative emotions. The emotion regulation strategy “Reappraisal” describes an antecedent-focused behavior, whereby a person is cognitively changing upcoming situations to switch the emotional impact they might have. “Suppression” is a response-focused strategy, suppression behavior, or emotional expression following an experience (5, 36). The German version of the ERQ (36) is composed of 10 items (6 items for reappraisal and 4 items for suppression) and is rated on a 7-point Likert scale ranging from 1 (“strongly disagree”) to 7 (“strongly agree”). Cronbach's alpha was α = 0.85 for “Reappraisal” and α = 0.73 for “Suppression.”

#### Personality Organization

The 16-Item *Inventory of Personality Organization* [IPO-16; German version by (37)] is a self-report measurement of deficits within personality structure. The questionnaire is theoretically grounded in Otto Kernberg's model of personality organization (38). The IPO-16 is composed of three subscales: (1) “Identity Diffusion,” which measures the integrity of the representations of oneself and others; (2) dominance of primitive defense mechanisms such as denial, splitting, projection, and dissociation (“Primitive Defense”); and (3) the capacity to differentiate between internal and external stimuli and to maintain the social shared reality (“Reality Testing”) (10). A total score of structural deficits can be generated with this instrument. The items are rated on a 5-point Likert scale ranging from 1 (“never”) to 5 (“always”). Internal consistencies for the subscales were acceptable ranging from Cronbach's α = 0.6 to α = 0.73. The total score showed acceptable internal consistency with a Cronbach's α = 0.74.

#### Adult Attachment

The *Adult Attachment Scale* [AAS; (39)] is a self-report questionnaire based on the assumption that early attachment experiences form relatively stable inner attachment working models that influence individual needs and behavior in later relationships (9, 10). The AAS consists of three subscales measuring anxiety about being rejected or unloved (“Anxiety”), comfort with closeness (“Close”), and comfort with depending on others (“Depend”). The German version of the AAS (40) is composed of 15 items (five items per subscale) and is rated on a 5-point Likert scale ranging from 1 (“strongly disagree”) to 5 (“strongly agree”). Cronbach's α for “Anxiety” was α = 0.77, that for “Close” was α = 0.84, and that for “Depend” was α = 0.77.

#### Substance Use

The *Alcohol, Smoking and Substance Involvement Screening Test* [ASSIST, (41)] is a standardized interview that is used to assess psychoactive substance use and related problems. This questionnaire measures lifetime use and substance-related symptoms of 10 substance groups, including tobacco, alcohol, cannabis, cocaine, amphetamines, inhalants, sedatives, hallucinogens, opioids, and “other drugs.” A global score was generated from the subscales (“frequency of drug use,” “craving to use drugs,” “(health, social, legal or financial) problems,” “failed expectations,” “expressed concerns by relatives and friends,” “failed attempt to cut down,” and “drug injection”). Cronbach's α for the subscales ranged between α = 0.67 and α = 0.75. The total score showed an excellent internal consistency with Cronbach's α = 0.9.

### Data Analysis

SPSS 27.0 was used for data management, descriptive statistic, bivariate correlations, and multiple hierarchical regression. In a first step, age and gender were included in the regression as control variables. The total score of the ASSIST was induced as a second step into the multiple hierarchical regression as a control variable. Finally, the global score of Personality Organization and the three scales “Depend,” “Close,” and “Anxiety” of the adult attachment scale were incorporated in a third step.

## Results

### Demographics and Sample Characteristics

The investigated sample consisted of 299 young adults (222 females, 74.2%). The participants ranged in age from 18 to 35 years (M = 22; SD = 3.81). A total of 51 (17.1%) participants reported a university degree as their highest educational level, 244 (81.6%) participants reported a high school degree, 3 (1%) participants declared a completed apprenticeship as their highest educational level, and one (0.3%) participant absolved compulsory education. Concerning the current relationship status, 156 (52.2%) reported to be single, 134 (44.8%) were in a relationship, 3 (1%) were married, 1 (0.3%) person was divorced, and five (1.7%) participants preferred not to share information about their relationship status. In terms of the question, which kind of substance was ever consummated, 227 (75.9%) participants reported to have consummated tobacco, 292 (97.7%) alcohol, 198 (66.2%) cannabis, 45 (15.1%) cocaine, 57 (19.1%) amphetamines, 23 (7.7%) inhalants, 47 (15.7%) sedatives, 53 (17.7%) hallucinogens, 19 (6.4%) opioids, and 28 (9.4%) “other drugs.” Since ASSIST is a standardized tool for estimating the severity of drug abuse and the resulting need for therapeutic intervention, the following groups were formed based on the results: (1) No intervention is needed; (2) Short-term intervention is advised; (3) Intensive treatment is advised. Participants were predominantly in the “no intervention is needed” group (tobacco: 56.9%; alcohol: 68.9%; cocaine: 93.6%; cannabis: 69.9%; amphetamines: 93.6%; inhalants: 99%; sedatives: 93.3%; hallucinogens: 94%; and opioids: 97%).

### Correlations

As normal distribution was not given for any of the examined variables, Pearson and Spearman correlations were both calculated. The changes in the correlations were negligible, and changes in correlation appearance/disappearance only were observed for correlations not concerning the dependent variables “Suppression” and “Reappraisal.” As shown in Table 1, bivariate correlations between the examined variables suggested that the ER strategy “Suppression” was significantly positively related to structural deficits in “Personality Organization” (*r* = 0.16; *p* < 0.01), anxiety about being rejected or unloved (“Anxiety”; *r* = 0.15; *p* < 0.05), and “Gender” (*r* = 0.19; *p* < 0.01), and significantly negatively related to comfort with closeness (“Close”; *r* = −0.46; *p* < 0.01) and comfort with depending on others (“Depend”; *r* = −0.36; *p* < 0.01). Furthermore, the ER strategy “Reappraisal” showed a significant positive correlation with “Depend” (*r* = 0.24; *p* < 0.01) and “Age” (*r* = 0.12; *p* < 0.05), and a significant negative correlation with structural deficits in “Personality Organization” (*r* = −0.15; *p* < 0.01) and “Anxiety” (*r* = −0.20; *p* < 0.01). In addition, structural deficits in “Personality Organization” showed an association to all three scales measuring adult attachment (“Anxiety,” *r* = 0.59; “Close,” *r* = −0.36; “Depend,” *r* = −0.50; all *p* < 0.01), as well as to “Substance Use” (*r* = 0.36; *p* < 0.01) and “Age” (*r* = −0.14; *p* < 0.05). “Substance Use” was moreover related to the attachment dimension “Depend” (*r* = −0.15; *p* < 0.01). Within the attachment scales, “Anxiety” showed a relation to “Gender” (*r* = −0.18; *p* < 0.01) and “Age” (*r* = −0.14; *p* < 0.05). “Depend” was associated with “Gender” (*r* = 0.13; *p* < 0.05) and “Age” (*r* = 0.13; *p* < 0.05). All attachment scales were correlated with each other (for all *p* < 0.01). Additionally, Bonferroni correction was conducted in order to control for α inflation. Hereby, the originally significant correlations between “Personality Organization” and “Reappraisal,” “Suppression” and “Anxiety,” “Depend” and “Substance Use,” “Gender” and “Depend,” as well as all statistically relevant correlations with “Age,” disappeared.

**Table 1:** Correlations among examined variables: Emotion regulation, personality organization, attachment styles, substance use, age, and gender.

**Variable**	**1**	**2**	**3**	**4**	**5**	**6**	**7**	**8**	**9**
1. Suppression	-								
2. Reappraisal	0.03	-							
3. PO	0.16**	−0.15**^a^	-						
4. Close	−0.46**	0.04	−0.36**	-					
5. Depend	−0.36**	0.24**	−0.50**	0.58**	-				
6. Anxiety	0.15*^a^	−0.20**	0.59**	−0.28**	−0.53**	-			
7. SU	0.07	−0.09	0.36**	−0.11	−0.15**^a^	0.08	-		
8. Gender	0.19**	0.05	−0.10	0.05	0.13*^a^	−0.18**	0.07	-	
9. Age	0.69	0.12*^a^	−0.14*^a^	0.04	0.13*^a^	−0.14*^a^	0.10	0.11	-
M or n	3.38	4.44	2.07	2.79	3.26	2.06	24.52	222	22
SD or %	5.07	7.28	0.57	4.26	3.80	3.92	31.54	74.2	3.81

*N = 299; Gender was coded as 0 = female and 1 = male*;

* *p < 0.05, and*

** *p < 0.01*;

a *non-significant after Bonferroni correction*.

*PO, structural deficits in “Personality Organization”; SU, “Substance Use.”*

### Multiple Hierarchical Regression

#### Suppression

The ASSIST was inserted into the stepwise regression, even though no correlations with ER could be found, because the ASSIST correlated with the IPO and some scales of the AAS.

Although some correlations had to be classified as non-significant after performing a Bonferroni correction, the originally selected predictor variables were adhered to. This decision is based on the fact that the Bonferroni procedure can be considered very conservative. In addition, most corrections were observed regarding the “Age” variable, which was still considered of interest mainly due to the explorative nature of the study.

However, ANOVA of the models, created through stepwise regression including, firstly, age and gender, secondly, the total score of the ASSIST, and thirdly, the global score of the IPO and the three scales of the AAS, showed that all three models predicted reliably the dependent variable “Suppression” [*F*_1(2.296)_ = 6.58; *F*_2(3.295)_ = 4.88; *F*_3(7.291)_ = 16.05; all *p* < 0.01]. The model summary, however, showed that model 3 explained 26.1% of the variance in “Suppression” and therefore accounted for the most variance. Furthermore, inserting the ASSIST did not show any significant change in *F*.

Regarding the coefficients of model 3, “Gender” and the scales “Close” and “Depend” showed significant results (all *p* < 0.01). “Close” showed the greatest effect on “suppression” (β = −0.38), followed by “Gender” (β = 0.23) and “Depend” (β = −0.18). Table 2 displays the results of the stepwise regression.

**Table 2:** Multiple hierarchical regression model for ER strategy “Suppression.”

	**Variable**	***B***	**β**	***p***	***Adjusted R*** ^**2**^	**St. error of estimate**
Step 1				**0.00**	0.036	1.24
	Gender	0.57	0.2	**0.00**		
	Age	−0.03	−0.09	0.12		
Step 2				**0.00**	0.038	1.24
	Gender	0.55	0.19	**0.00**		
	Age	−0.03	−0.1	0.09		
	SU	0.00	0.07	0.23		
Step 3				**0.00**	0.261	1.09
	Gender	0.67	0.23	**0.00**		
	Age	−0.02	−0.07	0.20		
	SU	0.00	0.02	0.78		
	PO	−0.13	−0.06	0.40		
	Close	−0.57	−0.38	**0.00**		
	Depend	−0.30	−0.18	**0.01**		
	Anxiety	0.01	0.01	0.93		

*N = 299; gender was coded as 0 = female and 1 = male; PO, structural deficits in “Personality Organization”; SU, “Substance Use”; Dependent Variable, ERQ “Suppression”; ER, “Emotion Regulation.” Bold values mean significant predictor*.

#### Reappraisal

ANOVA of the models, created through stepwise regression including, firstly, age and gender, secondly the total score of the ASSIST, and thirdly, the global score of the IPO and the three scales of the AAS, showed that models two and three significantly predicted the dependent variable “Reappraisal” [*F*_2(3.295)_= 2.70*, p* < 0.05;*F*_3(7.291)_= 4.10*; p* < 0.01]. The model summary showed that model three explained 6.8% of the variance in “Reappraisal” and therefore accounted for the most variance. Furthermore, checking the coefficients of models two and three, only age had an impact in model two (β = 0.13; *p* < 0.05), but lost its significant effect (β = 0.09; *p* > 0.05) as the IPO and AAS scales were inserted. Regarding the coefficients of model three, the variable “Depend” (β = 0.26) showed a significant result (*p* < 0.01). “Close” (β = −0.14) was found to be significant by tendency but will not be further interpreted. Table 3 displays the results of the stepwise regression.

**Table 3:** Multiple hierarchical regression model for ER strategy “Reappraisal.”

	**Variable**	***B***	**β**	***p***	***Adjusted R*** ^**2**^	**St. error of estimate**
Step 1				0.10	0.009	1.21
	Gender	0.10	0.04	0.53		
	Age	0.04	0.11	**0.05**		
Step 2				**0.05**	0.017	1.20
	Gender	0.12	0.04	0.46		
	Age	0.04	0.13	**0.03**		
	SU	−0.00	−0.11	0.06		
Step 3				**0.00**	0.068	1.18
	Gender	0.01	0.00	0.96		
	Age	0.03	0.09	0.13		
	SU	−0.00	−0.08	0.21		
	PO	0.05	0.03	0.75		
	Close	−0.19	−0.14	0.05		
	Depend	0.41	0.26	**0.00**		
	Anxiety	−0.15	−0.10	0.19		

*N = 299; Gender was coded as 0 = female and 1 = male; PO, structural deficits in “Personality Organization”; SU, “Substance Use”; Dependent Variable, ERQ “Reappraisal”; ER, “Emotion Regulation.” Bold values mean significant predictor*.

## Discussion

In this study, it was intended to investigate the influence of adult attachment patterns, personality organization, and substance use regarding the two ER strategies “Suppression” and “Reappraisal.” As revealed by stepwise regression analysis, we observed that for both strategies, adult attachment seems to have the most predictive value out of the examined variables. People who show more comfort with closeness and with depending on others tend to inhibit less often the (mimic, verbal, or gestural) expression of their feelings than people who show less secure attachment patterns in both the “Suppression” and the “Reappraisal” domains. Furthermore, we found “Gender” to play a crucial role in the selection of one specific ER strategy. Here, women tend to make a greater use of inhibiting the expression of their feelings (“Suppression”). In line with this finding, women show higher scores in the personality dimension Agreeableness (42, 43). Furthermore, it can be assumed in the light of gender role theories on affect that women use more self-focused, internalized activities in response to negative affect (44), resulting in the use of less expressive strategies. Gender, comfort with closeness, and comfort with depending on others accounted for over 26% of variance in “Suppression” of emotional expression. In comparison, findings concerning the ER strategy of “Reappraisal” show eminently less contribution. Only ~7% of variance in “Reappraisal” could be explained through the examined variables. The findings suggest that the capacity to depend on others comes along with higher use of strategies, which cognitively change the appraisal of situations and therefore their emotional impact.

Moreover, in this study, no significant results were found for ER with neither attachment “Anxiety” nor deficits in “Personality Organization.” In the case of “Reappraisal,” research supports the assumption that active adaptive strategy use shows weaker relationships with psychopathology than the use of maladaptive strategies (45). As attachment “Anxiety” and deficits in “Personality Organization” pose both characteristics of psychopathological behavior, this might cause the absence of significant results and furthermore explains the small variance in emotional “Reappraisal” caused by the examined variables. Another explanation could be that the score for structural deficits in “Personality Organization” and all three scales of the Adult Attachment Scale showed to be intercorrelated. Therefore, controlling for attachment, the absent significance of “Personality Organization” for “Suppression” as well as “Reappraisal” supports the assumption that attachment could play a more pronounced role in these specific emotion regulation strategies than personality organization. In this context, it could be hypothesized that attachment behavior measured by the AAS—similar to “Reappraisal” and “Suppression”—is linked to higher levels of mental processing than “Personality Organization” measured by the IPO, as the latter concept is focused on more severe levels of psychopathology (37, 46). Therefore, adult attachment could be more connected to higher brain functions such as ER. Finally, no connection whatsoever could be found between Substance Use and ER. This result could be due to the nature of the sample. Participants reported predominantly very little use of substances. Even for tobacco and alcohol, except for a small percentage, participants reported a rather mild use, where no therapeutic intervention was indicated.

### Limitations of the Study

Even though this study was carefully planned and conducted, it has some limitations, which must be taken into consideration for drawing a conclusion from the results. The substantially higher percentage of participating women must be mentioned as well as the comparatively high educational level of the participants. These demographics confine the representativity of the results. A higher educational level is attended with a reduced risk of substance abuse [e.g., (47)] or psychopathology [e.g., (48)]. It could also be theorized that the educational level influences the selection of emotion regulation strategies. Therefore, a more balanced sample regarding the educational level would have been advantageous. Furthermore, some of the correlations showed changes after applying a Bonferroni correction and have to be interpreted cautiously. Also, conducting research online has advantages, like an easier way to approach participants, a good manageability of data, and a greater sense of anonymity, but it also holds some possible disadvantages. For example, the environment in which the study is conducted is not controlled. Therefore, participants might be less attentive while filling in the questionnaires or they might get easily distracted. Also, the absence of an investigator-in-charge might evoke participants to fill in the questionnaires less thoroughly. Furthermore, two very specific emotion regulation strategies have been taken into account. A broader approach might have produced deeper insight into the broad field of emotion regulation. Finally, there is research supporting the hypothesis that emotion beliefs change emotion regulation (49). Whether one perceives emotions as good or bad, and controllable or uncontrollable might dictate the choice of emotion regulation strategies and it would have been interesting to examine emotion beliefs as well.

## Conclusion

Despite its limitations, this study contributes to a better understanding of how attachment, personality organization, and substance use might be connected to emotion regulation. The results suggest that attachment behavior particularly helps to explain the different selection of strategies in emotion regulation.

## Data Availability Statement

The raw data supporting the conclusions of this article will be made available by the authors, without undue reservation.

## Ethics Statement

The studies involving human participants were reviewed and approved by University of Graz. The patients/participants provided their written informed consent to participate in this study.

## Author Contributions

PB, XV, and H-FU conceptualized the study. PB and XV collected the data. PB analyzed and interpreted the data. PB and H-FU drafted the manuscript. JF and H-FU critically reviewed it. All authors gave their final approval of the manuscript.

## Conflict of Interest

The authors declare that the research was conducted in the absence of any commercial or financial relationships that could be construed as a potential conflict of interest.

## Publisher's Note

All claims expressed in this article are solely those of the authors and do not necessarily represent those of their affiliated organizations, or those of the publisher, the editors and the reviewers. Any product that may be evaluated in this article, or claim that may be made by its manufacturer, is not guaranteed or endorsed by the publisher.
